# Empagliflozin Increases Short-Term Urinary Volume Output in Artificially Induced Syndrome of Inappropriate Antidiuresis

**DOI:** 10.1155/2017/7815690

**Published:** 2017-12-20

**Authors:** Julie Refardt, Bettina Winzeler, Fabian Meienberg, Deborah R. Vogt, Mirjam Christ-Crain

**Affiliations:** ^1^Department of Endocrinology, Diabetology and Metabolism, University Hospital Basel, Basel, Switzerland; ^2^Clinical Trial Unit, Department Clinical Research, University of Basel and University Hospital Basel, Basel, Switzerland

## Abstract

**Objective:**

Syndrome of inappropriate antidiuresis (SIADH) is the predominant cause of hyponatremia, but treatment options are unsatisfying. SGLT2 inhibitors increase urinary glucose excretion with concomitant osmotic diuresis. We therefore hypothesized SGLT2-inhibitors as a novel treatment for SIADH.

**Design:**

Double-blind placebo-controlled randomised crossover study in 14 healthy volunteers.

**Methods:**

We induced an artificial SIADH model by administration of desmopressin and overhydration. Afterwards, empagliflozin 25 mg or placebo was given in random order. The main outcomes were total urinary excretion, glucosuria, and the area under the curve (AUC) of serum sodium concentration. Outcome measures were obtained 2–8 hours after administration of study drug.

**Results:**

14 participants (64% males), BMI 23 kg/m^2^ (±2.4), aged 28.6 years (±9), completed the study. Empagliflozin led to significantly increased total urinary excretion (579.3 ml (±194.8) versus 367.3 ml (±158.8); treatment effect 158 ml (CI 48.29, 267.74), *p* = 0.017) due to glucosuria (74.18 mmol (±22.3) versus 0.12 mmol (±0.04); treatment effect (log scale) 2.85 (CI 2.75, 2.96), *p* < 0.001). There was no difference in the AUC of serum sodium concentration (treatment effect 0.2 (CI −7.38, 6.98), *p* = 0.96).

**Conclusion:**

In our SIADH model, empagliflozin increased urinary excretion due to osmotic diuresis. Due to the short treatment duration, serum sodium levels remained unchanged. Real-live studies are needed to further examine empagliflozin as a new treatment for SIADH.

## 1. Introduction

The syndrome of inappropriate antidiuresis (SIADH) is the predominant cause of hyponatremia and is characterized by an imbalanced secretion of the antidiuretic hormone arginine vasopressin (AVP) [1–3]. The impaired AVP regulation leads to a reduction of free water excretion with following hypotonic hyponatremia [4, 5]. Therapeutic options, aside from treating the underlying disease, depend upon the onset of hyponatremia and severity of the symptoms and include primarily fluid restriction or hypertonic saline infusion [4, 6]. Alternative treatment options with loop diuretics, administration of oral urea, or vasopressin receptor antagonists (vaptans) are discussed controversially in the literature [4, 6, 7]. Despite those options, there are a considerable number of patients who do not sufficiently respond to treatment [7], making additional therapy necessary.

Empagliflozin is a sodium glucose cotransporter 2 (SGLT2) inhibitor, which has become a valuable treatment option for type 2 diabetes. The SGLT2 is expressed in the proximal tubule and reabsorbs approximately 90 percent of the filtered glucose [8, 9]. The inhibition of SGLT2 results in pronounced glucosuria with subsequent enhanced water excretion by osmotic diuresis [10]. This mechanism is of major interest in view of new therapeutic options in case of impaired water excretion as in patients with SIADH.

As patients with SIADH are usually older with several comorbidities and multiple medications [11, 12], studies evaluating new treatment options are difficult to interpret. We therefore created an artificial SIADH model in healthy volunteers via administration of desmopressin i.v. and overhydration. We hereby aimed to study the effects of the SGLT2 inhibitor empagliflozin in healthy volunteers in artificially induced SIADH with focus on urinary volume excretion, glucosuria, and change of serum sodium level.

## 2. Subjects and Methods

### 2.1. Study Design and Subjects

We performed a prospective double-blind, placebo-controlled randomised crossover study at the University Hospital Basel, Switzerland, from March to June 2016. The local ethics committee (EKNZ 2015-00024) as well as the national agency for the authorisation and supervision of therapeutic products (swissmedics 2016 DR 2031) approved the study protocol and study medication. The trial was registered at Clinicaltrials.gov (number NCT02729766).

Written informed consent was obtained from 15 healthy volunteers. They had no history of any chronic diseases; renal and hepatic impairment, thyroid dysfunction, and adrenal insufficiency were excluded through laboratory measurements. Besides oral anticonception in all females, participants were on no medication during the study period.

### 2.2. Procedures

**Figure 1:**
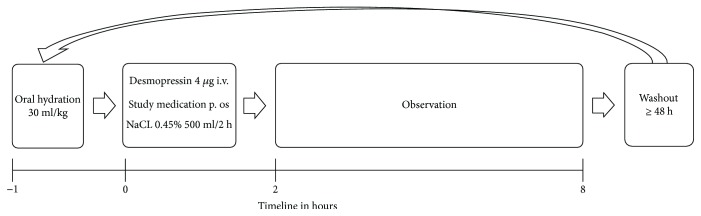
Schematic figure of the study procedure; h = hours.

The procedure and different timepoints are explained schematically in Figure 1.

Each subject underwent two study days receiving empagliflozin or placebo in randomized order with a washout period of at least 48 hours in-between. They reminded fasting after midnight and were admitted to our clinical trial unit between 6.30 and 7 a.m. No food was allowed until the end of the observation period. Drinking was only permitted during the oral hydration phase.

On arrival (timepoint −1), clinical symptoms attributed to hyponatremia (vertigo, headache, thirst, nausea, and malaise; visual analogue scale (VAS) 0–10), clinical parameters including body weight, blood pressure, and heart rate as well as blood and urinary parameters were evaluated and thereafter regularly throughout the study day.

After voiding the bladder, participants were asked to drink 30 ml water per kg body weight in one hour (corresponding to 2200 ml in average), additionally replacing fluid loss 1 : 1 if urinary excretion exceeded 100 ml within one hour.

After one hour (timepoint 0), desmopressin 4 *μ*g i.v. was injected, immediately followed by the application of the study drug (empagliflozin 25 mg or placebo) per os. Because empagliflozin and placebo tablets were not identical in colour and shape, participants were blindfolded and the administration was carried out by an independent doctor from the endocrine department who did not belong to the study team. The study team was blinded concerning treatment allocation of the participants. Afterwards, an infusion of NaCl 0.45% 500 ml over 2 hours was started (end of infusion = timepoint 2).

The primary observation phase was between timepoints 2 and 8 (=total 6 hours). At the end of the observation period, participants received a small meal (sandwich), and monitoring was maintained for 2 additional hours. Participants were discharged if they were in a clinically good condition and the serum sodium level was above 130 mmol/l.

### 2.3. Laboratory Measurements

Concentrations of sodium, glucose, creatinine, urea, uric acid, and osmolality were measured at different timepoints in serum and urine. The fractional excretion of urea and uric acid was estimated by the formula: fractional excretion (*U*_*y*_ × *P*_creatinine_/*U*_creatinine_ × *P*_*y*_), where *U* = urinary, *P* = plasma, and *y* = substance to be calculated.

Blood samples for serum copeptin, MR-proANP, NT-proBNP, renin, and aldosterone determination were drawn in a supine position, immediately centrifuged, and stored at −70°C for batch analysis. Serum copeptin and MR-proANP levels were measured with a commercial sandwich immunoluminometric assay (B.R.A.H.M.S CT-proAVP LIA, Thermo Scientific Biomarkers, Hennigsdorf, Germany) [13, 14]. The copeptin assay had a 0.4 pmol/l lower detection limit and <5% (<10%) intra-assay (interassay) coefficients of variation for concentrations > 2.0 pmol/l (>2.5 pmol/l). The MR-proANP assay had a lower detection limit of 6.0 pmol/l. Aldosterone and renin were measured with a standard chemiluminescent immunoassay (CLIA) with a reference range of 32–654 pmol/l and 1.7–23.9 ng/l, respectively, in a supine position. NT-proBNP was measured with a standard electrochemiluminescence immunoassay (ECLIA) with a reference range of <127 ng/l for males and <177 ng/l for females.

### 2.4. Statistical Analyses

The final analysis set included a total of 14 subjects who completed the whole procedure. One subject withdrew consent during the oral water hydration phase and therefore had to be excluded from further analysis.

The primary endpoint was the area under the curve (AUC, calculated by the trapezoid rule) of the serum sodium concentration between 2 and 8 hours after treatment; glucosuria and urinary volume excretion were further efficacy outcomes of primary interest. All other measurements were defined as secondary outcomes.

Possible symptoms of hyponatremia were averaged over the observation period and compared between empagliflozin and placebo using a paired Wilcoxon's signed rank test. This test was also used for the analysis of secondary outcomes at specific timepoints between empagliflozin and placebo.

The final outcomes evaluating the observation period between timepoints 2 and 8 were analysed using linear mixed effects models. The models included the corresponding measurement (timepoint 0 for body weight, urinary volume excretion, AUC serum sodium concentration, serum−/urinary osmolality, serum glucose, natriuresis, glucosuria, FEurea, FEuricacid; timepoint −1 for copeptin, MR-proANP, NT-proBNP, aldosterone, and renin) as covariate, treatment, treatment-sequence (i.e., empagliflozin-placebo versus placebo-empagliflozin) and their interaction as predictors (fixed effects), and subject as a random effect. Restricted maximum likelihood (REML) was used. Point estimates with 95% confidence intervals based on normal approximation and *p* values based on Satterthwaite's approximation are reported. Further, the least-squares means (i.e., the covariate-adjusted model predictions) for each treatment arm are given with 95% confidence intervals. Total glucosuria was log10 transformed in order to meet the assumptions of normally distributed errors. Patient characteristics are summarised as frequencies and percentages or as mean ± one standard deviation.

Analyses were performed using the statistic program R, version 3.3.1 [15]. All tests were two sided with *p* values < 0.05 considered to indicate statistical significance.

## 3. Results

**Figure 2:**
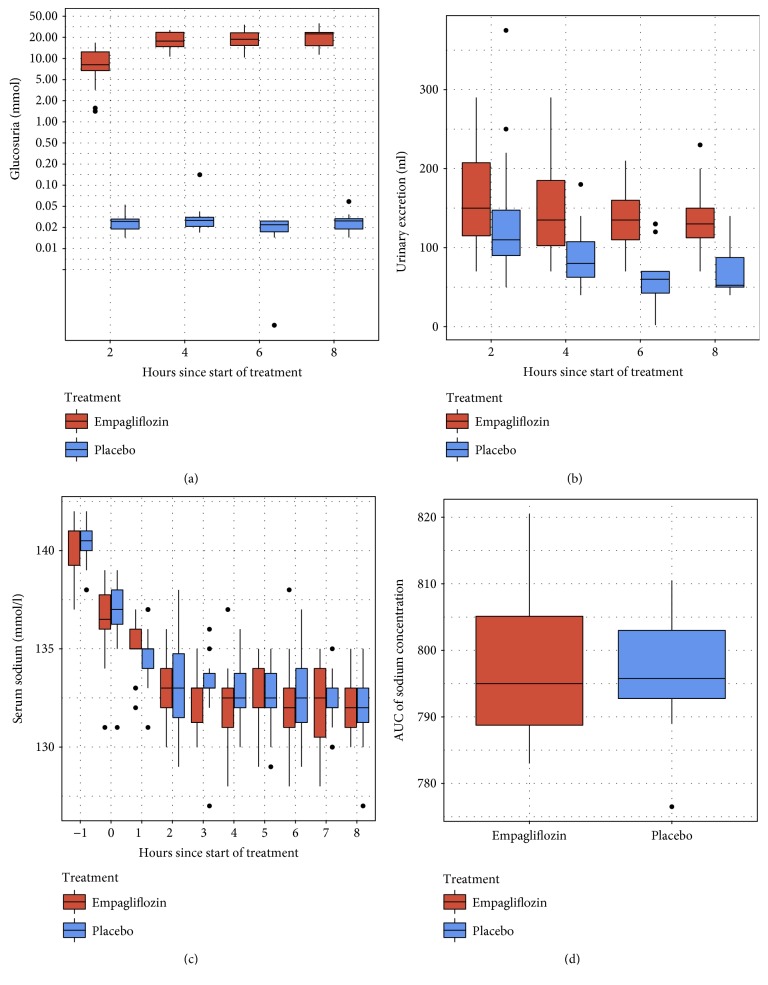
Efficacy outcomes according to treatment (empagliflozin versus placebo); the thick line indicates the median, the box indicates the interquartile range, and the whiskers include all points within the range of 1.5x the interquartile range. (a) Trajectory of glucosuria (note that *y*-axis is on log scale). (b) Trajectory of urinary excretion. (c) Trajectory of serum sodium. (d) Area under the curve (timepoints 2 to 8) for serum sodium concentration.

The main results are shown in Tables 1 and 2 and Figure 2.

14 participants (9 male (64%)) aged 28.6 years (±9) with a mean body mass index of 23.1 kg/m^2^ (±2.4) completed the study. Laboratory values on arrival were balanced on both study days; in particular, there was no difference in serum sodium level (empagliflozin 140 mmol/l (±1.5) versus placebo 140 mmol/l (±1.3), *p* = 0.9). Through the oral water load, serum and urinary sodium and osmolality decreased at timepoint 0, again with no difference between the two groups. Urinary volume excretion also remained similar (empagliflozin 202 ml (±98) versus placebo 229 ml (±142), *p* = 0.85).

At timepoint 2, the artificial SIADH had been induced resulting in hypotonic hyponatremia (overall: serum sodium 133 mmol/l (±2.0); serum osmolality 269 mmol/kg (±3.2)), elevated urine osmolality (overall: 502 mmol/kg (±138.7)), and natriuresis (overall: urine sodium 83 mmol/l (±39.5)). At this time, the incipient effect of empagliflozin was already noticeable, with a significant increase in glucosuria (empagliflozin 9.5 mmol (±5.6) versus placebo 0.0 mmol, *p* = 0.001).

Glucosuria under empagliflozin was maintained during the observation period between timepoints 2 and 8 (total glucosuria 74.18 mmol (±22.3) versus 0.12 mmol (±0.04); treatment effect (on log scale) 2.85 (CI 2.75, 2.96), *p* < 0.001) with no difference in plasma glucose (empagliflozin 4.1 mmol/l (±0.5) versus placebo 4.5 mmol/l (±0.3); treatment effect −0.16 mmol/l (CI −0.48, 0.17) *p* = 0.367). This led to a significantly increased diuresis under empagliflozin with a total urinary excretion of 579 ml (±194.8) versus 367 ml (±158.8) in the placebo group (treatment effect 158 ml (CI 48.29, 267.74) *p* = 0.017).

No difference was noted in serum sodium levels as well as in the change of the serum sodium concentration (AUC) between empagliflozin and placebo during the observation period (AUC serum sodium concentration 0.2 (CI −7.38, 6.98), *p* = 0.96). There was also no significant difference in total natriuresis calculated according to the excreted volume (empagliflozin 83.3 mmol (±42) versus placebo 64.4 mmol (±41); treatment effect 8.54 mmol/l (CI −14.19, 31.27), *p* = 0.5) however with an increase in the empagliflozin group towards the end of the observation period (Figure 2).

Fractional excretion of urea and uric acid did not differ between the two groups until timepoint 2 but increased significantly under empagliflozin by the end of the observation period (FE_urea_ empagliflozin versus placebo 42.9% (±9.3) versus 30.6% (±9.0); treatment effect 8.49% (CI 2.56, 14.41), *p* = 0.016; FE_uricacid_ empagliflozin versus placebo 10.9% (±4) versus 6.4% (±2.3); treatment effect 3.93% (CI 1.01, 6.86) *p* = 0.022).

The course of the different biomarkers was similar in both groups, with a decrease of copeptin, aldosterone, and renin and an increase of MR-proANP and NT-proBNP due to the induction of the artificial SIADH. Two copeptin values in the empagliflozin group had to be excluded from the analysis due to extraordinary high values (15.2 and 30.6 pmol/l) at timepoint 8, probably due to vomiting.

There was no difference in the average rating of clinical symptoms attributable to hyponatremia (Table 2). However, more adverse events were reported under empagliflozin: 6 versus 2 participants required pain medication against headache, 2 versus none suffered from acute vomitus, and 1 participant had acute transient hypoglycaemia of 2.7 mmol/l.

## 4. Discussion

The main finding of our study is that compared to placebo, the SGLT2 inhibitor empagliflozin leads to significantly increased water excretion in healthy volunteers with artificially induced SIADH. This is a novel finding, as according to available data in healthy volunteers and type 2 diabetic patients, no difference in total urine output when compared to placebo has been described [8, 16, 17]. The enhanced water excretion is promoted by osmotic diuresis as reflected by the increased glucosuria but also due to a direct modulation of the uric acid transporter [18]. Osmotic diuresis is well described in patients with type 2 diabetes under treatment with empagliflozin [9]; however, we here show for the first time that this effect also works in SIADH—a condition with a strong antidiuretic impact. The assumption that osmotic diuresis is the cause of the elevated urinary volume excretion is also supported by the increase of fractional urea and uric acid secretion [19]. As SIADH is characterised by pathological water retention, the acquaretic properties of empagliflozin may be effective as shown for other therapeutic strategies targeting water excretion [4, 6] vasopressin receptor antagonists (vaptans) [20] and oral urea [21, 22]. However, vaptans are expensive and bear the risk of sodium overcorrection, whereas oral urea is usually not well tolerated. Empagliflozin could be a safe and effective alternative. Given that our participants were not allowed to eat anything during the study, the therapeutic effect of empagliflozin could arguably be even more pronounced in real-life situation as the amount of glucose excreted in urine depends on the level of glycaemia [9].

Serum sodium levels did not differ between the two treatment arms at the end of the observation period. The lack of an effect of empagliflozin on serum sodium levels is possibly due to the short observation period of only six hours and the consequently relative small difference in urinary volume excretion of 200 ml. An extrapolation of those results to one day with further increase in total urinary volume excretion and thereafter change in serum sodium levels is tempting, but highly speculative. Nevertheless, keeping the pharmacodynamics of empagliflozin in mind with increasing urinary glucose excretion before reaching a plateau in the first 24 hours [16], this assumption cannot be excluded. Previous studies in healthy volunteers and diabetic patients are of limited value in regard of serum sodium levels [23, 24], as those populations do not suffer from free water retention which is a characteristic for SIADH [2].

A longer treatment period to detect a difference in serum sodium levels may also be needed because of the initial increase in natriuresis described under treatment with SGLT2 inhibitors [25]. The transient nature of this effect was shown by a recent study in type 2 diabetic patients with standardised food-, fluid-, and salt-intake and treatment with empagliflozin 25 mg for 5 days [26]. Natriuresis increased after the first empagliflozin dosage and normalized after multiple dosing [26]. Empagliflozin outcome studies including poorly controlled type 2 diabetic patients showed no events of hyponatremia, which also argues against a persistent natriuretic effect [27].

Because of the glucosuria and transient natriuresis under empagliflozin, calculation of the free water clearance and the electrolyte-free water clearance was not helpful to estimate the potential effect of empagliflozin on serum sodium levels.

There was an increased rate of adverse events under treatment with empagliflozin compared to placebo. The majority of reported symptoms are most likely due to the acute effects of hyponatremia; an aggravation through administration of empagliflozin is possible. This would argue against the use of empagliflozin in acute hyponatremia. As however most patients with SIADH suffer from chronic hyponatremia which is often clinically less symptomatic [11], the tolerability of empagliflozin should be tested in patients with chronic hyponatremia.

Some additional findings of our study are noticeable. First, the role of copeptin as a stress marker [28–30] can nicely be illustrated with our results: the levels of the two participants with vomitus were extraordinarily elevated despite persisting hypotonic hyponatremia. Second, there was an episode of transient mild symptomatic hypoglycaemia (2.7 mmol/l) despite intact liver function in one of the participants. This has so far not been described in studies evaluating the effect of empagliflozin in healthy volunteers [17]. As the hypoglycaemia occurred after a prolonged fasting period of 17 hours, the combination of urinary glucose loss and fasting may have led to the symptomatic decrease in glucose in this lean patient.

The following limitations have to be mentioned. First, the effect of empagliflozin was measured in an artificial SIADH model, which can never fully mirror the complex pathomechanisms of sodium and fluid homeostasis in clinical reality. Also, this was a small study involving 14 healthy participants who received only one dose of treatment and who do not represent the usually comorbid elderly patients with SIADH. However, our crossover design allowed us to study the physiological reaction without any confounding factors. Second, the observation time of the treatment effect was limited to six hours, making it difficult to extrapolate the results to a longer treatment phase. Unfortunately, the observation period in the proposed model cannot be prolonged due to side effects described above.

The strength of our study is its prospective double-blind, placebo-controlled randomised crossover design—with every participant being his own control, any physiological changes can be easily detected and analysed. Therefore, given the rapid and significant increase in urinary volume excretion under empagliflozin, we hypothesize that SGLT2 inhibitors could be a promising new treatment option for patients with SIADH.

In conclusion, in healthy volunteers with artificially induced SIADH, empagliflozin increased volume excretion due to osmotic diuresis. Due to the short treatment duration, serum sodium levels remained unchanged. Additional studies in real-live setting are needed to further examine the possible role of empagliflozin as a new treatment option for SIADH.

## Figures and Tables

**Table 1:** Clinical and laboratory variables shown as mean (standard deviation) according to the different timepoints.

	−1		0		2		8		Treatment effect	*p*
	Empagliflozin	Placebo	Empagliflozin	Placebo	Empagliflozin	Placebo	Empagliflozin	Placebo	Empagliflozin versus placebo	
*Clinical evaluations*										
Blood pressure (mmHg)	122/76 (13/9)	125/78 (14/8)	125/82(17/8)	129/85 (14/9)	124/75 (13/7)	124/76 (14/8)	127/71 (16/9)	126/75 (16/8)	—	
Pulse (bpm)	62 (10)	64 (10)	58 (9)	57 (10)	58 (8)	59 (10)	61 (8)	60 (9)	—	
Weight (kg)	75.6 (14)	75.7 (13.1)	77.5 (14.1)	77.6 (13.4)	77.6 (14.1)	77.9 (13.4)	77 (13.9)	77.3 (13.2)	0.08 (−0.18, 0.33)	0.556
Urinary volume excretion (ml)	—	—	202 (98)	229 (142)	165 (69)	140 (88.5)	579 (195)^∗^	367 (159)^∗^	158 (48.29, 267.74)	**0.017**
*Laboratory values*										
Serum										
Sodium (mmol/l)	140 (1.5)	140 (1.3)	136 (2.1)	137 (2.1)	133 (1.6)	133 (2.3)	132 (1.5)	132 (2)	0.2 (−7.38, 6.98)^∗∗^	0.960
Osmolality (mmol/kg)	286 (2.9)	286 (4.7)	278 (4)	281 (6.7)	269 (3.3)	269 (4)	270 (6.2)	267 (3.5)	−0.61 (−3.91, 2.68)	0.721
Glucose (mmol/l)	4.8 (0.4)	5.1 (0.5)	4.7 (0.3)	4.7 (0.4)	4.5 (0.3)	4.8 (0.2)	4.1 (0.5)	4.5 (0.3)	−0.16 (−0.48, 0.17)	0.367
Urine										
Sodium (mmol/l)	108 (38)	106 (41)	51 (30)	50 (36)	80 (39)	86 (41)	188 (27)	237 (59)	—	
Total natriuresis (mmol)	—	—	—	—	14.1 (9.7)	12.9 (12.4)	83.3 (42)	64.4 (41)	8.54 (−14.19, 31.27)	0.498
Osmolality (mmol/kg)	678 (249)	586 (240)	302 (175)	260 (178)	536 (121)	468 (151)	823 (60)	879 (98)	−48.75 (−116.78, 19.28)	0.185
Glucose (mmol/l)	0.3 (0.2)	0.3 (0.1)	0.1 (0.1)	0.1 (0.1)	67.5 (46.7)	0.2 (0.1)	174.8 (42.1)	0.4 (0.1)	—	
Total glucosuria (mmol)	—	—	—	—	9.5 (5.6)	0.0 (0.0)	74.18 (22.3)	0.12 (0.04)	2.85 (2.75, 2.96)^∗∗∗^	**<0.001**
Fractional excretion										
FE urea (%)	44.6 (6.2)	44.3 (8.3)	49.9 (5.3)	50.4 (6.9)	40.8 (7.6)	32.2 (8.5)	42.9 (5.3)	30.6 (9.0)	8.49 (2.56, 14.41)	**0.016**
FE uric acid (%)	5.8 (1.4)	5.2 (2.2)	6.4 (1.7)	6.2 (1.9)	10.2 (1.9)	8.6 (2.4)	10.9 (2.9)	6.4 (1.7)	3.93 (1.01, 6.86)	**0.022**
Biomarkers										
Copeptin (pmol/l)	5.9 (3.7)	5 (1.9)	—	—	3.1 (0.7)	3.1 (0.7)	3.3 (1.8)	2.6 (0.5)	0.36 (−1.09, 1.82)	0.63
NT-proBNP (ng/l)	27.1 (14.0)	31.7 (18.6)	—	—	30.3 (17.4)	34.4 (21.5)	50.9 (32.2)	50.5 (33.0)	4.94 (−13.00, 22.87)	0.59
MR-proANP (pmol/l)	48.7 (11.8)	49.2 (16.7)	—	—	61.7 (14.8)	59.3 (15.1)	63.1 (18.9)	56.8 (8.9)	6.92 (−5.94, 19.77)	0.31
Aldosterone (pmol/l)	371.2 (253.1)	314.7 (154.1)	—	—	175.6 (93.3)	154.1 (59.0)	128.5 (60.1)	86.7 (23.6)	29.73 (−14.53, 73.99)	0.21
Renin (ng/l)	14.3 (9.4)	21.2 (26.7)	—	—	6.3 (5.3)	8.4 (7.4)	4.9 (5.1)	3.6 (3.2)	1.93 (−1.55, 5.40)	0.29

^∗^Timepoint 8 = cumulative excretion during observation period; ^∗∗^AUC sodium observation period; ^∗∗∗^values on log scale; FE: fractional excretion. Treatment effect: estimated effect sizes (95% confidence interval) empagliflozin versus placebo, *p* values according to mixed effects models.

**Table 2:** Number of participants who experienced symptoms attributable to hyponatremia during the study with the according average score of symptom severity on visual analogue scale in brackets (range 0 = no symptoms to 10 = severe symptoms).

Symptoms	Empagliflozin	Placebo	*p*
Thirst	9 (2.4)	7 (2.9)	0.51
Headache	10 (2.4)	11 (1.7)	0.75
Nausea	7 (3.1)	6 (3.0)	0.67
Vertigo	6 (2.6)	3 (2.5)	0.50
General malaise	7 (2.8)	5 (3.0)	0.94
